# Editorial: Chemotherapy in esophageal cancer

**DOI:** 10.3389/fonc.2022.1098838

**Published:** 2022-12-20

**Authors:** Jiang Chen, Hao Liu, Prasanna K. Santhekadur

**Affiliations:** ^1^ Department of General Surgery, Sir Run Run Shaw Hospital, Zhejiang University, Hangzhou, China; ^2^ Nanfang Hospital, Southern Medical University, Guangzhou, China; ^3^ Department of Biochemistry, Center of Excellence in Molecular Biology & Regenerative Medicine, Jagadguru Sri Shivarathreeshwara (JSS) Medical College, Jagadguru Sri Shivarathreeshwara (JSS) Academy of Higher Education and Research, Mysore, Karnataka, India

**Keywords:** chemotherapy, esophageal cancer, drugs, metastasis, cancer

Esophageal cancer is the cancer of esophagus and is one of the most deleterious cancers and sixth most common cause of carcinogenesis related mortality worldwide (1). Although incidence rates of esophageal cancer vary based on geographic locations, the major causes for this malady includes tobacco chewing, smoking, excess alcohol consumption, practicing western sedentary lifestyle and dietary habits and associated obesity (2) (3). These causes activate various signalling pathways at cellular levels and initiate esophageal cancer development (4) (Elliott and Reynolds). Although there are many treatment options for esophageal cancer this issue mainly focused on chemotherapy. In this special issue we received many research and review articles on chemo and associated therapies to treat this cancer and two of these review articles by Luo et al and Huang et al sheds light on the role of PI3K/Akt/mTOR signalling pathway in development and pathophysiology of esophageal squamous cell carcinoma (ESCC). They also suggested that either PI3K/Akt/mTOR or their downstream eukaryotic translation initiation factors (eIFs) may act as potential therapeutic targets to treat this disease. A study by Xiao et al discussed the possible brain metastasis of ESCC and concluded that development of characteristics of brain metastases is rare in these patients and suggested that local or specific territorial (locoregional) treatment is associated with improved overall survival. Another *in silico* study by Zhao et al predicted the overall survival and benefits of chemotherapy using Deep Learning (DL)-based protein features in gastric cancer and they also demonstrated the advantages of DL-based workflow in gastric cancer molecular subtyping along with its possible therapeutic application. Study by Yang et al suggested that the esophageal cancer patients who are intolerable to surgery or who are under the impact of old age or geriatric patients (aged ≥80 years), should prefer chemoradiotherapy (CRT) as a preferable treatment option compared to other therapies.

A retrospective, propensity score-matched short-term study by Feng et al discussed the clinical efficacy of neoadjuvant chemotherapy (NACT) combined with Laparoscopic gastrectomy (LG) for locally advanced adenocarcinoma of the esophagogastric junction and concluded that these combined therapies does not increase the risk of postoperative morbidity and mortality when compared with LG alone (https://pubmed.ncbi.nlm.nih.gov/34660265/). A population study associated SEER analysis by Yang et al revealed that ESCC subjects with organ specific metastasis other than liver or bone have more benefits from local ablative treatment (LAT) with systemic chemotherapy. A study by Kermani et al concluded that in ESCC patients’ predictive or anticipating value of endoscopic results, observations, impressions and biopsy after neoadjuvant CRT are insufficient for assessing overall complete pathological response after neoadjuvant treatment and they also suggested that additional methods are required for overall assessment of the treatment and its impact. A phase II randomized study by Wang et al compared the preoperative concurrent CRT versus NACT or neoadjuvant chemotherapy for locally well-developed later stage gastric cancer (LAGC) patients in a single center-based data. In contrast to this report another study by Zheng et al compared the side effects and effectiveness of chemical drugs Lobaplatin-based versus Cisplatin-based adjuvant chemotherapy data from multicenter study and based on their analysis, Lobaplatin plus docetaxel might be a better choice of drug for adjuvant chemotherapy particularly for ESCC. Finally, A systematic review and meta-analysis by Xia et al. concluded that consolidation chemotherapy (CCT) after Concurrent chemoradiotherapy (CCRT) significantly increases over all long-term survival and disease progression-free survival of patients with nonsurgical esophageal cancer and could provide them astonishing overall survival benefits.

Finally, these elegant research and review articles increased the current knowledge and added additional information about benefits and drawbacks of different therapeutic options for patients with advanced esophageal cancer.

## Author contributions

All authors listed have made a substantial, direct, and intellectual contribution to the work and approved it for publication.
